# Enhancing species discovery and description in algal turfs: A case study in the green alga *Pseudoderbesia* (Bryopsidales)

**DOI:** 10.1111/jpy.70122

**Published:** 2026-01-20

**Authors:** Amelia Hastings, Chiela Cremen, Myles Courtney, Yuqun Du, Heroen Verbruggen

**Affiliations:** ^1^ Melbourne Integrative Genomics, School of BioSciences University of Melbourne Parkville Australia; ^2^ CIBIO, Centro de Investigação em Biodiversidade e Recursos Genéticos, InBIO Laboratório Associado, Campus de Vairão Universidade do Porto Vairão Portugal

**Keywords:** DNA, genomics, microscopic, nomenclature, species discovery, taxonomy, turf algae

## Abstract

Algal turfs are assemblages consisting of small marine green, brown, and red algae on the scale of millimeters to a few centimeters. Due to their small size, they have been less intensively studied by macroalgal taxonomists, and they also fall outside the scope of microalgal taxonomists, who tend to focus on smaller, often unicellular, taxa. They often have a rather simple structure and a tendency to converge onto similar morphologies with creeping and upright axes. Because of all of this, there is a substantial amount of undocumented algal biodiversity in turfs, as has been shown in several molecular surveys. Our aim in this paper was to explore some integrative taxonomic methods that could help accelerate the discovery and description of very small turf species. We focused on *Pseudoderbesia*, a genus of extremely small green algae from the family Bryopsidaceae. We used a combination of multifocal imaging of field‐collected samples, microsample genomics, and culturing to document the *Pseudoderbesia* biodiversity from Heron Island on the Great Barrier Reef. Algorithmic species delimitation based on *rbc*L and *tuf*A marker genes indicated that likely six (possibly five) species exist in *Pseudoderbesia*, but only two have been described. We have formally described the two species discovered at Heron Island as *P. luxurians* and *P. epilithica*. The latter was described using a multifocal image as the holotype, following an exception to the nomenclatural code for microscopic algae. We have justified this choice extensively, both based on an interpretation of the code and on the broader conceptual need to name newly discovered species, facilitating their use in science, conservation, and policy.

**Abbreviations**
ASAP: Assemble Species by Automatic Partitioning
GMYC: Generalized Mixed Yule Coalescent
HTS: high‐throughput sequencing
MCMC: Markov chain Monte Carlo
ML: maximum likelihood
PTP: Poisson Tree Processes

## INTRODUCTION

Turf algae refers to a diverse group of small algae up to several centimeters tall that are often observed in intertidal and subtidal marine environments (Connell et al., 2014). On coral reefs, they cover roughly 40% of the substrate (Harris, 2015) and contribute to the ecosystem as food for marine herbivores (Ledlie et al., 2007) and sites of nutrient cycling (El‐Khaled et al., 2021; Haas et al., 2011). Climate change, overfishing, and high nutrient input all contribute to increasing the competitive advantage of turf algae over corals (Johnson et al., 2017; Zaneveld et al., 2016).

Turf algae can be difficult to identify by morphology alone; they suffer from a “low morphology problem,” wherein the simplicity of their morphology can lead to morphologically indistinguishable species (Van Oppen et al., 1996; Verbruggen, 2014). DNA‐based methods can help distinguish species in such difficult groups and have become the primary species delimitation criterion in many algal studies (Leliaert, Verbruggen, Vanormelingen, et al., 2014). Díaz‐Tapia and Verbruggen (2024), for instance, described eight separate species within the *Polysiphonia scopulorum* species complex (now moved to the genus *Bryocladia*), some of which were morphologically indistinguishable. This is not an unusual situation, and many other studies have shown that morphologically defined turf algal species contain substantial levels of cryptic biodiversity (e.g., Díaz‐Tapia et al., 2020; Leliaert et al., 2009; Verbruggen, Vlaeminck, et al., 2009).

The rate of species description of turf algae is restricted by several factors. Foremost, they are small. They are often overlooked by seaweed taxonomists that focus on larger taxa. Sorting through turfs under the stereo microscope can be quite laborious, and this situation is made worse by the fact that several species often grow intermingled, making the sorting of individuals extra difficult. Second, their small stature restricts what can be achieved with the limited material available. For seaweed integrative taxonomy, it is common practice to preserve a portion of the thallus in silica gel and another in liquid preservative for anatomical observations The remainder of the specimen is pressed and becomes the herbarium voucher. This is not feasible with turf algae because they are too small, and especially if one considers that many look‐alike species are lurking in turfs, then it is advisable to be cautious when grouping multiple individuals together in a single collection. Sometimes, culture strains can be isolated to grow more material, permitting more types of analyses and preservation methods.

For this study, we focused on *Pseudoderbesia*, a siphonous green alga that occurs in tropical turfs. It has a simple morphology of dichotomously branching upright siphons arising from a horizontal “runner” siphon, with short rhizoid siphons attaching the algae to the substrate (Calderón & Schnetter, 1991). *Pseudoderbesia* is particularly small: Most thalli observed in nature are just a few millimeters tall and can often not be readily observed in the field; rather, they are seen when inspecting macroalgae or other substrates with a stereomicroscope.

Only two species have been described: *Pseudoderbesia arbuscula* from Colombia (Calderón & Schnetter, 1991) and *P. eckloniae* from Western Australia (Huisman & Verbruggen, 2020). *Pseudoderbesia* has also been collected from the Canary Islands (Calderón & Schnetter, 1991) and Rhodes, Greece (Leliaert, Verbruggen, D'Hondt, et al., 2014), but these samples were assigned to a species, as the researchers felt there was not enough information about the genus to do this. Through environmental sequencing, *Pseudoderbesia* DNA sequences have also been recovered from Louisiana (Sauvage et al., 2016) and Hawai'i (Wade & Sherwood, 2017).

Our goal in this paper was to explore methods to accelerate the discovery and description of turf algae, with a focus on the biodiversity of *Pseudoderbesia* on Heron Island (Great Barrier Reef) and the evaluation of species diversity within the genus more broadly.

## MATERIALS AND METHODS

We carried out two expeditions to Heron Island on the southern Great Barrier Reef to collect *Pseudoderbesia*. Due to the extremely small size of individual *Pseudoderbesia* algae, we were very limited in our options of what to do with that material, as the individual filaments were too small to preserve for both DNA and morphological work. On the first expedition in February 2017, our approach consisted of imaging field‐collected *Pseudoderbesia* filaments in the field laboratory and then sacrificing the whole filament in a DNA extraction for downstream high‐throughput sequencing (HTS), an approach we have called microsample genomics for the purpose of this paper. Photographs were taken at various focal depths with the built‐in camera of a Leica EZ4 HD stereo microscope, and a focus stack of those images was generated in Affinity Photo v.2 (Serif, Nottingham, United Kingdom). For a second expedition in April 2023, we focused on establishing cultures of the filaments, observing the material, and carrying out DNA extractions after some growth had occurred. Original and focus‐merged images were provided in the Zenodo repository associated with this study (https://doi.org/10.5281/zenodo.16568275).

Cultures were grown on a shaker in 50‐mL culture flasks in F/2 media (Guillard, 1975; Guillard & Ryther, 1962) at 26°C at a 12:12 h light:dark cycle (16 μmol photons · m^−2^ · s^−1^). Small pieces of uncontaminated algae were periodically moved into new containers, and vitamin‐less media was used at times to manage bacterial growth. At the completion of the project, all remaining culture material was killed in 100% ethanol and deposited in the University of Melbourne Herbarium (MELU).

DNA was extracted with the modified Doyle and Doyle (1987) CTAB extraction protocol described in Cremen et al. (2016). For some samples, we modified this by including an overnight incubation step at 65°C, adding a third chloroform:isoamyl alcohol extraction, extending the isopropanol precipitation step overnight, and not performing RNAse treatment. Illumina libraries were prepared using a VAHTS universal DNA kit and sequenced on the NovaSeq platform (150 bp PE). Reads were filtered and trimmed with fastp v0.24.0 (Chen, 2023) using the default settings and assembled with Megahit v1.2.9 (Li et al., 2015), and the *rbc*L and *tuf*A gene sequences, which have been used as DNA barcodes in *Pseudoderbesia* and related genera (Leliaert, Verbruggen, D'Hondt, et al., 2014; Verbruggen, Ashworth, et al., 2009), were extracted from the assemblies. For some samples, we used polymerase chain reaction (PCR) to amplify the *rbcL* gene and Sanger‐sequenced the *rbc*L gene. The PCR used a 50‐μL volume with 25 μL of NEB Taq 2× Master Mix, 1 μL of each of forward and reverse primers at a concentration of 10 μM, 2 μL of template DNA at a concentration of 10 μM, and 21 μL of H_2_O. The primers used were 295F (5′ ATGGATGGTGCTATTYTMGTRG 3′) and 1114R (5′ TTCGATCTCCAGGCATKACC 3′). Primers were designed with the help of Primer 3 v2.3.7 (Koressaar & Remm, 2007; Kress & Erickson, 2008; Untergasser et al., 2012) in Geneious Prime 2025.2 (Dotmatics, 2025). An initial denaturation for 30 s at 95°C was followed by 35 cycles of denaturation (20 s at 95°C), annealing (30 s at 47°C), and extension (55 s at 68°C), after which a final extension was carried out for 5 min at 68°C. In addition to the material we obtained from the Great Barrier Reef, we also sourced an additional specimen of *P. eckloniae* from Rottnest Island that was subjected to Illumina sequencing as described above. The *rbc*L and *tuf*A gene sequences for all samples were submitted to Genbank (accessions PV999908‐22), and chloroplast genome contigs were extracted from the assemblies and provided in the Zenodo repository. Raw read data were submitted to the sequence read archive (study PRJEB101882).

Multiple sequence alignments were made in Geneious Prime using MAFFT v7.490 (Katoh & Standley, 2013). In addition to our Australian sequences, the alignments also included all *tuf*A and *rbc*L gene sequences for *Pseudoderbesia* from Genbank and a *Pseudoderbesia rbc*L gene sequence from Hawai'i supplied by Rachael Wade (Wade & Sherwood, 2017). The outgroup sequences were *rbc*L and *tuf*A gene sequences of the species *Lambia antarctica* and *Bryopsis plumosa*, two other genera in the Bryopsidaceae family.

Three species delimitation algorithms were run on the single‐gene alignments: Assemble Species by Automatic Partitioning (ASAP; Puillandre et al., 2021), Generalized Mixed Yule Coalescent (GMYC; Fujisawa & Barraclough, 2013), and Poisson Tree Processes (PTP; Zhang et al., 2013). The ASAP algorithm was run using its command‐line version with default parameters. For PTP analyses, we inferred maximum likelihood (ML) phylogenies of the single‐gene alignments with IQ‐Tree 1.6.12 (Minh et al., 2020) using the best model as determined by ModelFinder (Kalyaanamoorthy et al., 2017): TIM2 + F + Γ4 for *rbc*L and TIM2 + F + Γ4 for *tuf*A. Bootstrap values were determined using the package UFBoot2 (Hoang et al., 2017) with 1000 replicates. We inferred species boundaries with the command‐line version of bPTP. For GMYC, due to known optimization problems of this software when identical sequences are present (Verbruggen et al., 2025), we deduplicated identical sequences to retain only the longest copy, and ultrametric trees were created with BEAST v.1.10.4 (Suchard et al., 2018) with an uncorrelated log‐normal relaxed clock model (Drummond et al., 2006), a coalescent constant size tree prior (Kingman, 1982), and 1 million Markov chain Monte Carlo (MCMC) iterations. We derived a maximum clade credibility tree with median node heights, discarding the first half of the MCMC samples as burn‐in. Generalized Yule Mean Coalescent species delimitation (Fujisawa & Barraclough, 2013) was run on R v. 4.4.0 (R Core Team, 2021) using the package splits v.1.0‐20 (Ezard et al., 2021). The alignments used for this work are available in the Zenodo repository.

To obtain a phylogenetic tree containing all species in a single phylogeny, a concatenated alignment was created using the *rbc*L and *tuf*A gene sequences of a single sample of each species suggested by the species delimitation algorithms. Species for which only one of the genes was known were included using that single gene. An ML tree of the concatenated phylogeny was generated with IQ‐Tree 1.6.12 (Minh et al., 2020), using the TIM2 + F + I + G4 model as determined by the built‐in ModelFinder (Kalyaanamoorthy et al., 2017) and 500 standard bootstraps. The concatenated alignment is available in the Zenodo repository.

## RESULTS

From the 2017 expedition, we obtained *rbc*L and *tuf*A gene sequences of three *Pseudoderbesia* samples using our first approach (i.e., sacrificing the filament in DNA extraction after photographing it). On the 2023 collection trip, small *Pseudoderbesia*‐like algae filaments with dichotomous branching were observed at all collection locations we surveyed at Heron Island, growing on calcium carbonate rock as well as epiphytically on *Halimeda*, *Laurencia*, *Padina*, *Sargassum*, *Caulerpa*, *Turbinaria*, *Dictyota*, and coralline crustose algae. Of >70 filaments isolated in the field station, only a fraction survived transportation and successfully grew in culture. We were able to obtain *rbc*L and *tuf*A gene sequences for four cultured samples.

**FIGURE 1:**
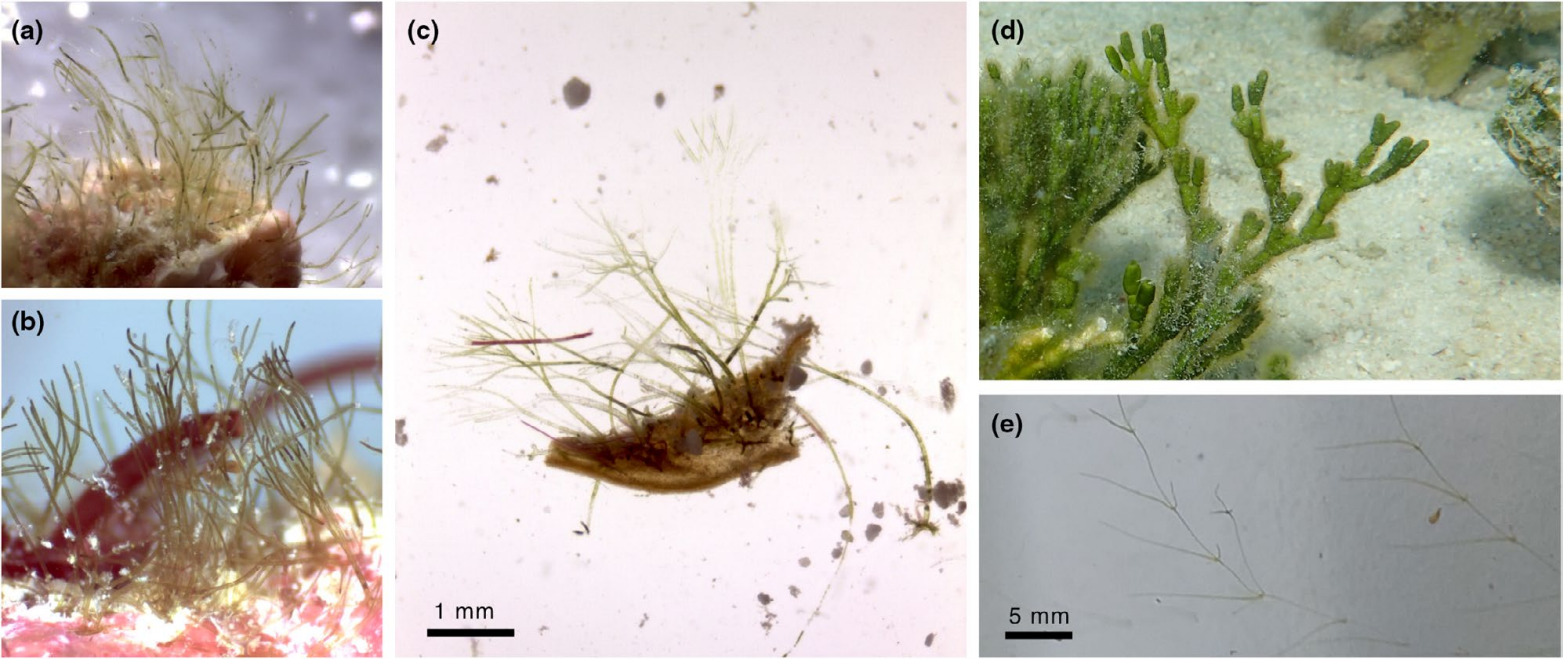
Field‐collected and cultured *Pseudoderbesia* specimens from Heron Island, Australia. (a) *Pseudoderbesia epilithica* HV6614a. (b) *Pseudoderbesia epilithica* HV6710 (holotype image). (c) *Pseudoderbesia luxurians* HV6621b. (d) *Halimeda cylindracea* covered in diverse epiphytes including *P. luxurians*. (e) *Pseudoderbesia luxurians* (strain MC37) growing in culture. Scale for (a) and (b) is the same as in (c). Note that the illustrations in panels (a–c) were generated by a focus merge of several images taken at different focal planes.

In field samples, a variety of morphologies were observed, with some specimens having finer, more flexible upright siphons while others were more rigid (Figure 1a–c). Multiple layers of dichotomous branching were present in many specimens. Culture sample morphology was observed as a horizontal “runner” siphon with regularly arising upright siphons, but we did not observe any dichotomous branching of the uprights in our cultures (Figure 1e) during the few months that we grew them. The internode length between upright siphons in the cultures was approximately 5 mm, whereas the upright siphons were approximately 8 mm tall. The rhizoid siphons occurred in pairs, growing from the runner siphon directly below and on either side of the upright siphon. The rhizoid siphons were approximately 1 mm long and attached the algae to the walls of the flask. The algae had a sparse growth habit (Figure 1e), especially compared to the growth habit shown in the environment (Figure 1a–c).

**FIGURE 2:**
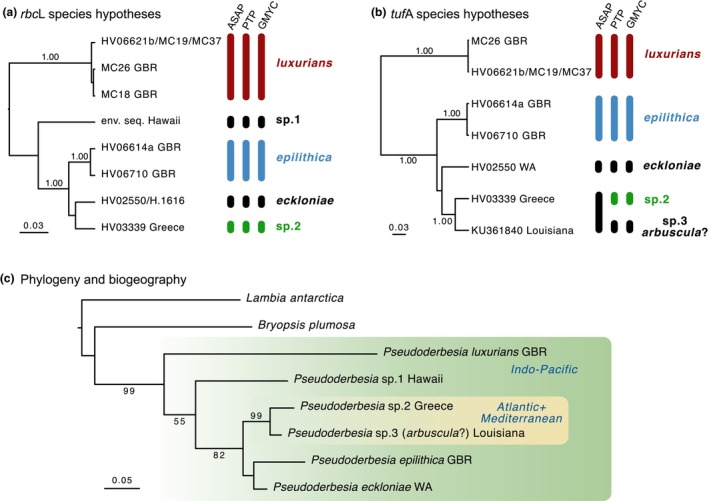
Molecular species delimitation and phylogeny of *Pseudoderbesia*. (a) Species hypotheses inferred from *rbc*L sequence data, with vertical bars to the right of the tree indicating species delimitation results from ASAP, PTP, and GMYC. The tree was inferred with BEAST after deduplication of identical sequences (shown separated by slashes, e.g., HV2550/H.1616). (b) Species hypotheses inferred from *tuf*A gene sequence data, with tree inference and indication of deduplicated sequences as for panel (a). (c) Maximum likelihood tree inferred from concatenated *rbc*L and *tuf*A gene sequences of *Pseudoderbesia* species. Scale bars on the phylogenies are in estimated numbers of substitutions per site. Branch support is indicated on branches when the posterior probability exceeds 0.95 or the bootstrap value reaches 50.

The assemblies of our micro‐sample genomics HTS data yielded draft chloroplast genomes, with the candidate contigs having ca. 212× coverage for HV06621 and ca. 50× coverage for HV06614 and HV06710. The combination of *rbc*L and *tuf*A gene sequences from previous work, the Sanger sequences of our cultures, and the candidate plastid contigs from our microsample genomics data was used to infer species boundaries. For the *rbc*L gene, the species delimitation analyses consistently recovered five species (Figure 2a). Only *Pseudoderbesia eckloniae* could be given an existing name with certainty, since one of the sequences was derived from the type specimen. The remaining species‐level lineages were two Great Barrier Reef species that we have described below, along with sp.1 from Hawai'i and sp.2 from Greece. In the *tuf*A gene, which was missing the Hawai'i entity but included an additional sequence from Louisiana, PTP and GMYC suggested five species, with ASAP suggesting four (Figure 2b). Four of these were also present in the *rbc*L data (*P. luxurians*, *P. epilithica*, *P. eckloniae*, and *P*. sp.2), and the fifth, from Louisiana, was designated as sp.3. The sequences from our 2017 and 2023 Heron Island expeditions originating from cultures and field‐extracted DNA (indicated with GBR on Figure 1a,b) clustered into two separate species: one consisting of two field‐extracted DNA sequences (HV06614a and HV06710) and the other with a combination of cultures (MC18,19,26,37) and field‐extracted DNA (HV06621b).

The phylogenetic analysis based on the concatenated *tuf*A and *rbc*L gene sequences resulted in a tree with relatively good support for many nodes (Figure 2c). A few other nodes, however, were less well resolved; for example, <50 for the grouping of the second new species and *P. eckloniae* and just 55 for the placement of *P*. sp.1 in the broader tree. These unstable branches may be to some extent due to the missing data for some species, with only the *rbc*L gene available for *P*. sp.1 and only *tuf*A for *P*. sp.3. It did seem clear from the concatenated tree and single‐gene topologies that the first new species below and *P.* sp.1 form relatively early‐branching lineages, while *P*. sp.2, *P*. sp.3, the second new species, and *P. eckloniae* appeared to have resulted from a more recent radiation.

## DISCUSSION

### Unrecognized biodiversity in *Pseudoderbesia*

Currently, the genus *Pseudoderbesia* is very understudied, with published papers detailing only four specimens worldwide, and two species formally described (Calderón & Schnetter, 1991; Huisman & Verbruggen, 2020; Leliaert, Verbruggen, D'Hondt, et al., 2014). Our results showed clear evidence for previously unrecognized biodiversity in *Pseudoderbesia*, with likely six (minimum five) species indicated in our molecular species‐delimitation results. All three methods of species delimitation agreed with each other on where the species boundaries lay in a majority of cases.

The *tuf*A gene sequences of the Greek and Louisiana samples, however, were split by PTP and GMYC into different species, whereas ASAP grouped them together as one species. To provide more context for these molecular species boundaries, we compared the distance between these hypothetical species to that between closely related species in more intensively studied Bryopsidales genera. The uncorrected distance (*p*‐value) between the Greek and Louisiana *Pseudoderbesia* samples was 5%. In *Caulerpa*, the divergences between pairs of closely related species recognized by Belton et al. (2019) were substantially less, ranging from 0.34% to 1.06% (Table S1). Similarly, in *Rhipilia*, the closely related but morphologically very distinct species *R. coppejansii*, *R. nigrescens*, and *R. orientalis* had uncorrected pairwise distances of 1.1%–1.4% for the *tuf*A gene (Verbruggen & Schils, 2012). These distance comparisons suggest that these two *Pseudoderbesia* sequences likely belong to separate species, which is how we have labeled them. However, we recognize the limitations of transferring distance thresholds across groups and the extremely low number of samples that have been sequenced in this part of the tree, so our decision should not be seen as the final word on this topic.

Although we largely relied on molecular sequences for species delimitation, an important aspect of our work was to document the field‐collected specimens. Our approach used multifocal stereomicroscope imaging, which resulted in detailed images that adequately captured the main morphological features of the tiny species we studied, allowing comparison with other species and playing a crucial role in typification (see below). Even though motorized microscopes and dedicated software applications for multifocal imaging are commercially available, we opted for a more portable and affordable version based on a Leica EZ4 HD compact stereo microscope with a built‐in digital camera, manually taking pictures at different focal depths. We used a commercial application for focus merging, as this was available to us via an institutional subscription, but many other software applications also have this functionality.

### Description of two Great Barrier Reef species

For the Great Barrier Reef samples, all molecular species delimitation methods consistently identified two clearly separated species. These were clearly two older lineages compared to the *Pseudoderbesia* sp.2 + *P*. sp.3 cluster, and both these Australian entities were well differentiated from other taxa. Hence, we formally describe the following two species:

***Pseudoderbesia luxurians* Verbruggen, sp. nov**.

**Description:** This species is primarily defined based on its *rbc*L and *tuf*A gene sequences, which are different from those of all other known *Pseudoderbesia* species as seen in phylogenetic analyses (Figure 2). Thalli have creeping and upright axes, attached to the substrate by means of elongated rhizoids. In field‐collected material, uprights were dichotomously branched and gradually tapered in thickness from the base to the tip of the thallus, 40–63 μm wide near the base, 32–36 μm near the middle of the thallus, and 10–15 μm when measured ca. 30 μm from the tips. Uprights were 2.5–3.9 mm tall.

**Holotype:** We designate metabolically inactive cultured material of MC26 as the holotype. The original material was collected by Myles Courtney on April 8, 2023, in Heron Island, Queensland, Australia, at a depth of ca. 1 m. Deposited in MELU.

**Etymology:** Named after the luxurious habit of the species

**Habitat:** Observed as an epiphyte on a variety of seaweeds including *Halimeda*, *Laurencia*, *Padina*, *Sargassum*, *Caulerpa*, *Turbinaria*, and *Dictyota*, growing in shallow‐water lagoon habitats

**Specimens examined:** HV06621b: Heron Island, February 26, 2017; epiphyte of *Padina* sp. MC18, MC19, and MC26: Heron Island, April 8, 2023; epiphytes of *Halimeda cylindricea.* MC37: Heron Island, April 10, 2023, epiphyte on *Halimeda cylindricea*.

***Pseudoderbesia epilithica* Verbruggen, sp. nov**.

**Description:** This species is primarily defined based on its *rbc*L and *tuf*A gene sequences, which are different from those of all other known *Pseudoderbesia* species as seen in phylogenetic analyses (Figure 2). Thalli have creeping and upright axes, attached to the substrate by means of elongated rhizoids. Siphons taper in diameter from their base, where they measure 30–55 (−65) μm to their tips (16–30 μm), with measurements near the center of the thallus 26–38 μm. The taper is less pronounced than in other *Pseudoderbesia* species, and the siphons end more bluntly than in other *Pseudoderbesia* species. Branching is relatively sparse. Uprights are 1.6–2.2 mm tall. Specimen HV06710 was larger than HV06614a in all dimensions, with the former measuring 1.6–2.2 mm tall, 35–55 (−65) μm at the base, 30–38 μm at the center, and 25–30 μm near the tips. The latter measured 1.8–2.0 mm tall, 30–45 μm at the base, 26–35 μm at the center, and 16–20 μm near the tips.

**Holotype:** The image of sample HV06710 (Figure 1b) serves as the holotype for this species. No physical specimen is available to serve as the holotype, since all material available to us was consumed in DNA extractions. The material was collected by Heroen Verbruggen on 3 March 2017, on Heron Island, Queensland, Australia, depth ca. 16 m.

**Etymology:** Named after the epilithic nature of the species

**Habitat:** This species grows on calcareous reef substrates, often on rubble covered in coralline crustose algae. Our two collections of the species were from deeper water on the reef slope.

**Specimens examined:** HV06614a: Heron Island, February 25, 2017, growing on rubble on a rubble slope, 18 m depth; HV06710: Heron Island, March 3, 2017, on crustose coralline algae growing on rubble, ca. 7 m depth

### Justification of nomenclatural decisions

We made the decision to describe *Pseudoderbesia epilithica* as a new species using an image rather than a voucher sample as a holotype. We realize this is likely to be a controversial decision that requires a rarely used rule of the International Code of Nomenclature for algae, fungi, and plants (Madrid Code; Turland et al., 2025). Article 8.1 indicates that “The nomenclatural type (see Art. 7.2) of a name of a species or infraspecific taxon is a holotype (Art. 9.1), lectotype (Art. 9.3), neotype (Art. 9.8), or conserved type (Art. 14.9), any of which may be supported by an epitype (Art. 9.9). Such a type is either a single specimen conserved in one herbarium or other collection or institution, or is a published or unpublished illustration (but see Art. 8.5; see also Art. 40.6 and Art. 40 Ex. 10).” Article 40.1 further specifies “Publication on or after 1 January 1958 of the name of a new taxon at the rank of genus or below is valid only when the type of the name is indicated.”

With regards to what this type should be, Article 40.6 is particularly relevant in our case: “For the name of a new species or infraspecific taxon published on or after 1 January 2007, the type indicated in accordance with Art. 40 may not be an illustration.” Based just on this text, the publication of *Pseudoderbesia epilithica* would be invalid, as we designate an illustration rather than a specimen as the holotype. Importantly, Article 40.6 includes the following exception: “An exception is permitted for names of non‐fossil microscopic algae and non‐fossil microfungi, for which the type may be an effectively published illustration if there are technical difficulties of specimen preservation or if it is impossible to preserve a specimen that would show the features attributed to the taxon by the author of the name.”

We rely on article 40.6 of the Madrid Code for the valid publication of *Pseudoderbesia epilithica*. Two key phrases—“microscopic algae” and “technical difficulties of specimen preservation”—require justification in our opinion. First, we argue that the species is a microscopic alga. The alga is a very fine filament, with a diameter of merely a few tens of micrometers, and the filaments are barely 2 mm tall. As such, it certainly cannot be recognized in the field, and it was only when rubble was collected and observed under a stereomicroscope that the filaments could be seen. Second, we argue that technical difficulties prevented specimen preservation. The amount of material available was so small that, using the DNA extraction methods we employed, all of the material was required in order to obtain sufficient DNA for low‐input library preparation. One may argue that the amount of material could have been increased by culturing. That is a valid argument; however, we attempted this during the 2023 expedition, when we also collected specimens resembling *P. epilithica* from rubble, yet our attempts resulted in failed algae growth and algae death despite *P. epilithica* having been incubated exactly like our successful *P. luxurians* cultures, indicating that *P. epilithica* presents technical difficulties for culturing. One may also argue that turf algae can easily be preserved as microscope slide preparations. That is true in most cases; however, these *Pseudoderbesia* samples were excessively small (2 mm) and did not allow for the creation of a permanent slide in addition to DNA extraction. This brings up the broader obstacle: Any future collection made of the same species in the same habitat will face this same dilemma. Either one can prepare a permanent slide, in which case the strain cannot be assigned to a molecular lineage with confidence, or one can perform DNA extraction and forego the preservation of a physical voucher. It is therefore not solely the lack of material for our collections that impedes a physical holotype; it is a feature of the species.

For *Pseudoderbesia luxurians*, the situation is more straightforward because in this case, we had a cultured specimen (now dead and dried) that served as the holotype. It must be noted that this culture is not representative of the appearance of the species in nature, as it exhibits a much more spread‐out habit and lacks branching of the uprights. The illustration in Figure 1c provides a better representation of the species growing in its natural environment.

### Biogeographic and morphological interpretations

The compiled data also showed that *Pseudoderbesia* appears to be spread across tropical and warm‐temperate habitats worldwide. The fact that the two Atlantic‐Mediterranean (Greece/ Louisiana) samples were nested within a larger Indo‐Pacific (Hawai'i/WA/GBR) clade (Figure 2c) suggests that the genus may have a tropical Tethyan origin, with the older Indo‐Pacific lineage giving rise to an Atlantic and Mediterranean lineage due to the closure of the Tethys Sea in the Middle East region during the Miocene (Bialik et al., 2019). From what we know of the species so far, it appears that individual species do not have a wide distribution, as every location has returned a separate species. Of course, as we only analyzed a relatively small number of *Pseudoderbesia* samples from a handful of locations, we cannot expect these phylogenies to offer a complete picture of the broader distribution of the genus, and more sampling is needed to evaluate this tropical Tethyan origin hypothesis. We have also considered that the sequence from Louisiana may represent *P. arbuscula* due to its proximity to where *P. arbuscula* was collected in Colombia by Calderón and Schnetter (1991). We have tentatively included this name in our phylogenies, although obtaining a sequence from the *P. arbuscula* type specimen (or new specimens obtained from the type locality) is needed to confirm this.

Leliaert, Verbruggen, D'Hondt, et al. (2014) pointed out that specimens of *Pseudoderbesia* have different rhizoid morphologies, either lobed or unlobed. *Pseudoderbesia arbuscula* from Colombia had lobed rhizoids surrounded by a mucilaginous layer (Calderón & Schnetter, 1991) whereas the specimen from Greece had lobed rhizoids without a mucilaginous layer (Leliaert, Verbruggen, D'Hondt, et al., 2014). Note that table 2 in the Leliaert, Verbruggen, D'Hondt, et al. (2014) paper indicated unlobed rhizoids for the Greek specimen, but this is a typographical error (Leliaert, pers. comm.), which the specimen images clearly have confirmed. The Canary Islands specimens, *P. eckloniae* and *P. luxurians*, have elongated rhizoids (Calderón & Schnetter, 1991; Huisman & Verbruggen, 2020), so this character on its own does not appear to be sufficient for morphological species identification.

We also compared the differing rhizoid morphology in this genus against our phylogeny. All the Indo‐Pacific species had unlobed rhizoids, whereas the specimen from Greece had lobed rhizoids. As we suggested above, it is possible that the Atlantic Louisiana sequence represents *Pseudoderbesia arbuscula*, which, as seen in the illustrations in Calderón and Schnetter (1991), has lobed rhizoids. In this case, lobed rhizoids may be a feature uniting the Atlantic‐Mediterranean clade, but clearly, more work is needed to confirm the Louisiana specimen as *P. arbuscula*.

The two Heron Island species, *Pseudoderbesia luxurians* and *P. epilithica*, differed in their habitats, with *P*. *luxurians* growing as an epiphyte on *Halimeda cylindricea*, *Padina*, and other corticated algae, whereas *P. epilithica* was observed on carbonate substrates. There also appeared to be differences in morphology between these two species. First, differing levels of tapering led to blunter siphon tips in *P. epilithica*, clearly shown by the larger siphon diameter measurements near the apices for *P. epilithica* (16–30 μm) than for *P. luxurians* (10–15 μm). Second, *P. luxurians* was larger than *P. epilithica*, the former nearly always exceeding 2 mm and branching many times, while the latter was extremely small, up to ca. 2 mm and not branching nearly as much. The two newly described species are much smaller than most other described species. For example, the specimens of *P. eckloniae* illustrated by Huisman and Verbruggen (2020) were 5.6–6.2 mm tall, and the field material reported for the Greek entity was 5 mm (Leliaert, Verbruggen, D'Hondt, et al., 2014). The heights reported for cultures of *P. arbuscula* by Calderón and Schnetter (1991) were (1.5–) 3.7 (−4.3) mm, which puts them in the same order of magnitude as *P. luxurians*, but that species differs in its rhizoid morphology (see above). In this context, it is important to also note the stark differences between the morphology of field‐collected and cultured materials. Cultures typically grow much more sparsely than the densely branched and carpet‐forming field‐collected samples (Figure 1e vs. Figure 1c,d). This limits the value of formal comparisons between the different species, as some have been documented predominantly from cultures (e.g., *P. arbuscula* and the Greek specimen), whereas others have been documented predominantly from field material (e.g., *P. eckloniae*, *P. luxurians*, and *P. epilithica*).

### Enhancing rates of species description

Species names are important: They are the cornerstone of most applications of species in society. But many species remain unnamed, limiting their inclusion in science and society. A large number of informal or provisional names for so‐called “dark taxa,” species known from sequence data but remaining unnamed and without formal descriptions, has been accumulating in the literature (De Clerck et al., 2012). Naming such species facilitates clear communication across disciplines, including conservation biology, natural resource management, aquaculture, and other industrial applications. Without names, these taxa remain invisible, stalling their integration into applied research, management, and policy. Moreover, deferring their formal recognition burdens future generations with unresolved taxonomy and lost knowledge.

We followed this philosophy in describing the two species, even though limited information was available for them. We acknowledge that, in principle, additional collections could be made of these species, and newer lower‐input library preparation techniques may allow for the preservation of a portion of a sample as a physical holotype for *Pseudoderbesia epilithica*; however, this line of reasoning encourages inaction. We have argued instead for a pragmatic approach: If the available evidence, both molecular and morphological, is substantial and robust, then formal species description is justifiable and should be encouraged. By embracing a data‐informed strategy, we can move taxonomy forward in a rigorous fashion, responsive to the realities of modern biodiversity science. Delaying the naming of taxa on the grounds that more complete data might eventually become available prioritizes idealism over progress, hindering the connection of these organisms to the broader scientific enterprise and facilitating their inclusion in conservation and policy, which is important for the turf algae that are gaining ground worldwide (e.g., Filbee‐Dexter & Wernberg, 2018; Johnson et al., 2017).

Of course, opinions on this may differ, and our conversations with algal nomenclature experts on the nomenclatural decisions in this paper have resulted in mixed reactions, from strong opposition to full agreement. We want to be clear that we are not in favor of playing it fast and loose with the nomenclatural rules just for the sake of getting names on lineages in trees. Whenever feasible, such as in the case of *Pseudoderbesia luxurians*, physical voucher deposition is the preferred solution, but we acknowledge that this is not always possible, especially for rare, small, and difficult‐to‐preserve organisms. For both our newly described species, we have provided extensive supporting data, including multi‐focal imaging to capture morphological detail and high‐quality DNA sequences, to ensure reproducibility and comparability as datasets continue to grow. Importantly, the species described here were supported by rich sequence libraries that will enable future extraction of alternative marker genes, as well as genome‐wide approaches to species delimitation, an emerging area in phycology (e.g., Bringloe et al., 2025; Verbruggen et al., 2025). These data‐rich taxa may ultimately be easier to integrate into future taxonomic syntheses than many older names tied to physical types from which no viable DNA can be obtained.

There have been interesting debates in the nomenclatural community about some proposals to allow the naming of taxa based on sequences as types (see, e.g., Thiele et al., 2023). Thus far, such proposals have not been successful (Turland et al., 2024). Among many concerns is that allowing naming basedon sequences as types would open the door for descriptions of many species that have never been directly observed, those from environmental sequencing, for example. That situation differs from the situation presented here, where an illustration has been nominated as the type, and extensive morphological information has been provided for the new species. We do refer to DNA sequences of holotypes as a central part of the descriptions of our species. We are of the opinion that in structurally simple organisms like *Pseudoderbesia*, wherein species show morphological overlap, DNA sequences are likely to be more informative about species boundaries and are more likely to remain informative as more new species are discovered.

This study's taxonomic and nomenclatural difficulties were eased by the limited treatment of *Pseudoderbesia* in the literature. Relatively few species had previously been described; hence, we could be fairly certain that the species we observed on the Great Barrier Reef were new. That said, some *Derbesia* species have relatively even dichotomous branching patterns, and they may turn out to be *Pseudoderbesia*, in which case the potential synonymy with the *Pseudoderbesia* species described here will need to be evaluated. In this spirit, we reached out to herbaria and colleagues to obtain material of those species. The type material of *D. fastigiata* in the University of Michigan Herbarium (MICH) was small and had very likely been formalin‐fixed so was unlikely to yield viable DNA. The type material of *D. padinae* is housed in the Bishop Museum (BISH), but destructive sampling was not permitted. We also tried to obtain the specimen of *D. attenuata*, illustrated in Littler and Littler (2000), which shows the same regularly dichotomous branching as *Pseudoderbesia*. The staff at the Smithsonian Institution (US) was able to find the original drawings, but these showed no mention of a specimen number, and no specimens of *D. attenuata* were located in their main inventory database. We also reached out to phycologists in Micronesia and Vietnam, hoping they may have had recent collections of *D. padinae* and *D. attenuata*, but that was not the case.

This type of due diligence could be substantially more challenging in genera with many names that have been described, particularly if there are many species for which no reliable reference material has been sequenced. Although we have advocated for the description of newly discovered species, we recognize the need for old names to be integrated into DNA‐based taxonomies to the extent that it is feasible. When comparison of morphological features has been insufficient for this purpose, approaches based on sequencing holotypes and/or recent collections from near the type locality of the species in question (topotypes) have been used to achieve this (e.g., Gabrielson et al., 2023; Huisman & Saunders, 2022). In other cases, pragmatic decisions have been made despite uncertainty about which molecular lineage is likely to match the type specimens, employing expert taxonomic judgment based on all the evidence available (e.g., Belton et al., 2014; Lagourgue et al., 2022; Sherwood et al., 2019).

Although our work significantly advances our knowledge of *Pseudoderbesia* biodiversity and distribution, it is likely that much more remains to be discovered. During just two short expeditions to a single location on the Great Barrier Reef, we observed two new species. The other data included in our trees suggest that other places have substantial additional biodiversity that needs to be put into a formal taxonomic framework. It will be particularly important to enhance knowledge of the genus in the Atlantic Ocean, where uncertainty remains about how many species should be recognized (Figure 2b) and where additional observations of *Pseudoderbesia* were made without molecular data (e.g., Canary Islands; Calderón & Schnetter, 1991). This is a crucial area for evaluating which entity should carry the name of the type species, *P. arbuscula*. Our conclusions about species in the Atlantic‐Mediterranean clade have been based on very small sample sizes, and small sample sizes have been known to reduce the performance of species‐delimitation methods (Ahrens et al., 2016). Broader sampling in this area will be needed for more conclusive results, and sequencing the type material for *P. arbuscula* from the Herbarium of the National University of Colombia in Bogotà (COL) may help in the assignation of this name to the correct lineage. Further discoveries will likely be made through expanded geographic sampling, as our data suggest that species appear to have restricted distributions. This is especially promising in the Indo‐Pacific, a vast and ancient region that remains relatively underexplored.

## AUTHOR CONTRIBUTIONS

**Amelia Hastings:** Data curation (equal); formal analysis (supporting); investigation (supporting); writing – original draft (equal). **Chiela Cremen:** Data curation (supporting); formal analysis (supporting); investigation (supporting); methodology (supporting); writing – review and editing (supporting). **Myles Courtney:** Data curation (supporting); methodology (supporting); writing – review and editing (supporting). **Yuqun Du:** Data curation (supporting); investigation (supporting); writing – review and editing (supporting). **Heroen Verbruggen:** Conceptualization (lead); formal analysis (lead); funding acquisition (lead); investigation (supporting); methodology (lead); project administration (lead); resources (lead); supervision (lead); writing – original draft (equal); writing – review and editing (lead).

## Supporting information


**Table S1.** Sequence divergences (uncorrected *p*‐values) of the *tuf*A gene between closely related *Caulerpa* species pairs.
