# Supplementary material for: Enhancing species discovery and description in algal turfs: A case study in the green alga Pseudoderbesia (Bryopsidales)
Source: J Phycol. 2026 Jan 20;62(1):222–33. doi: 10.1111/jpy.70122 (PMC12961170; doi:10.1111/jpy.70122)
Supplement: Supplementary file 1 — Table S1. Sequence divergences (uncorrected p‐values) of the tufA gene between closely related Caulerpa species pairs. [file JPY-62-222-s001.pdf]

Table S1. Sequence divergences (uncorrected p-values) of the *tuf*A gene between closely related *Caulerpa* species pairs.

**Species pair**	**samples**	**Genbank accessions**	**p-value**
remotifolia / *scalpelliformis*	GWS025195 / GWS015575	KF649920 / KF649898	1.06%
heterophylla / *muelleri*	AD-A? / AD-A92239	MH643881 / JN817643	0.63%
obscura / *alternans*	AD-A90909 / AD-A88966	JN817644 / KF649862	0.53%
cactoides / *corynephora*	AD-A74763 / PERTH08292590	JN817650 / JN817653	0.34%
